# Hot Deformation Behavior of a Ti-40Al-10V Alloy with Quenching-Tempering Microstructure

**DOI:** 10.3390/ma11060872

**Published:** 2018-05-23

**Authors:** Liang Cheng, Yi Chen, Guang Yang, Li Xie, Jiangtao Wang, Yalin Lu, Hongchao Kou

**Affiliations:** 1 School of Materials and Engineering, Jiangsu University of Technology, Changzhou 213001, China; chenyi@jsut.edu.cn (Y.C.); xlustb@163.com (L.X.); jxwjt@jsut.edu.cn (J.W.); luyalin@163.com (Y.L.); 2 Sunnywell (China) New Material Technology Co., Ltd., Changzhou 213000, China; 3 College of Mechanical and Electrical Engineering, Shaanxi University of Science and Technology, Xi’an 710021, China; yangguang_wxn@126.com; 4 State Key Laboratory of Solidification Processing, Northwestern Polytechnical University, Xi’an 710072, China; hchkou@nwpu.edu.cn

**Keywords:** TiAl alloys, deformation behavior, microstructure, micro-texture, recrystallization, grain boundary sliding

## Abstract

In this study, a Ti-40Al-10V alloy with quenching-tempering microstructure was prepared and was characterized by ultra-large β/B2 grains and submicrocrystalline γ laths within it. A definite Kurdjumov-Sachs orientation was identified between the β/B2 and γ phase. Isothermal compression tests were performed to examine the hot deformation behavior at various temperatures and strain rates. Based on the hyperbolic-sine equation, the deformation kinetics of the alloy were characterized by unexpectedly high activation energy (384 kJ/mol) and low stress exponent (2.25). For all the deformed samples, continuous dynamic recrystallization intensively occurred in the β matrix, accompanied by the simultaneous rotation of the γ laths. Moreover, a preferential orientation of <100>_β_ and <111>_γ_ parallel to the compression axis was observed for β and γ phase, respectively. With the decreasing strain rates, the grain boundary/interface sliding gradually became prominent, which resulted in some superplastic deformation features, e.g., intensive strain-induced grain growth and interface migration, enhancing “wetting” of the γ grain boundaries, continuous weakening/vanishing of the local texture, etc. Meanwhile, the temperature played an insignificant role in the hot deformation behavior. The deformation mechanism was discussed in detail based on the microstructural observations and deformation kinetics.

## 1. Introduction

γ-TiAl-based alloys possess superior high temperature mechanical properties and therefore are widely recognized as having the potential to be used in aero-engine industries [1,2,3]. However, they suffer from natural brittleness, which leads to difficulties in the wrought processes [1,4,5]. Hence, investigations regarding the hot deformation behavior of TiAl alloys are particularly important for the wrought routine design. In the past ten years, the hot deformation behavior of TiAl alloys has been extensively investigated by means of uniaxial compression at 900–1300 °C under constant strain rates in a range of 10^−4^–10 s^−1^ [1,6]. In general, such a parameter range results in the dislocation creep mechanism varying from power-law creep to power-law breakdown [6]. Then, the hyperbolic-sine law has been widely applied to describe the deformation kinetics:

(1) $$Z\equiv \dot{\varepsilon }\exp\left(Q_{\mathrm{app}}/RT\right)=A{\left[\sinh\left(\alpha \sigma \right)\right]}^{n},$$

where *Z* is the so-called Zener-Hollomon parameter, $\dot{\varepsilon }$ is the true strain rate, *Q*_app_ is the apparent activation energy, *A* and *α* are the material constant, *σ* is the true stress, and *n* is the stress exponent. In a recent review [6] as well as summarized by Appel et al. [1], one can find that for most TiAl alloys with (α_2_ + γ) as the major constituents, their stress exponents are in the range of 3–5 with a mean value of 4, and the average *Q*_app_ is about 370 kJ/mol which is close to the self-diffusion energy of Al in TiAl lattice (360 kJ/mol [7]). This indicates that during dislocation creep, for both the power-law creep and power-law breakdown regimes, the intragranular dislocation climb is responsible for the rate-controlling step, and the rate-controlling mechanism is the Al self-diffusion in the TiAl lattice.

Apparently, the hot deformation behavior is relatively simple to be clarified for (α_2_ + γ) TiAl alloys. However, the situation is quite curious for the V-rich TiAl alloys. It is well-recognized that the V element is a very strong β-stabilizer (1.6 times Nb element [8]), and hence generally there is noticeable β/B2 (B2 is the ordered form of β at low temperatures. Hereinafter we use “β” to denote both B2 and β) phase in the alloys. The β phase, owing to its soft nature at elevated temperatures, is believed to be beneficial for hot deformation of TiAl alloys [9]. However, as demonstrated by Kong et al. [10], the *Q*_app_ of Ti-43Al-9V and Ti-43Al-9V-0.3Y alloy were 577 kJ/mol and 451 kJ/mol, respectively. Such high values seem to be contradictory to the intuition that the β/B2 phase can decrease the apparent activation energy. More interesting conclusions can be drawn from the research results by Nobuki et al. [11]. In this study, they have prepared three TiAl alloys, namely, Ti-43.1Al-13.1V, Ti-39.2Al-9.3V, and Ti-43.6Al-5V, respectively with 29 vol %, 36 vol % and 10 vol % β/B2 phase. Hot tension at various temperatures and strain rates has been carried out to examine the hot deformation kinetics. The results revealed that all the alloys have impressively low *n* values (1.5–2), but more surprisingly, the activation energy values (484 kJ/mol and 426 kJ/mol) of the two alloys with excessive β/B2 phase were significantly larger than that of the β/B2-lean alloy (363 kJ/mol).

From the abovementioned background, one can note that the hot deformation behavior of V-rich TiAl alloys exhibits abnormal features such as low stress exponent and high activation energy. This point seems to be neglected till date and requires analysis in detail. In addition, our previous studies have demonstrated that the V-rich TiAl alloys can be readily heat-treated by the quenching-tempering (Q&T) process [12], and the obtained Q&T microstructure showed impressive mechanical properties from room to elevated temperatures [13]. However, the characteristics of the hot deformation behavior for such a microstructure are unknown and awaiting the reveal.

## 2. Materials and Methods

### 2.1. Materials Preparation and Compression Tests

**Figure 1:**
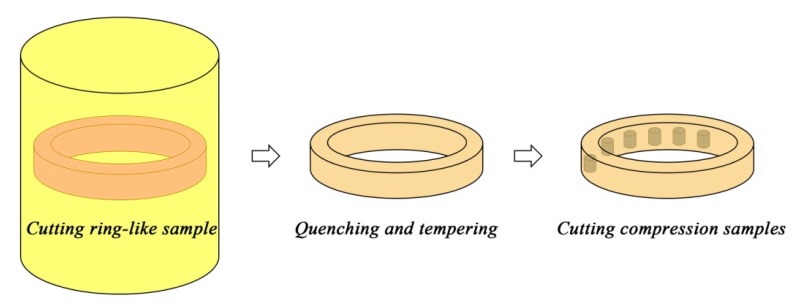
Schematic shows the preparation process of the samples for compression.

A Ti-38.89Al-9.62V ingot with a dimension of Φ90 × 420 mm was prepared by vacuum arc re-melting plus induction skull melting, and then hot isostatic pressed at 1250 °C/140 MPa for 4 h. A coaxial ring-like sample was cut from the ingot, as shown in Figure 1. Then the sample was annealed at 1350 °C (single β phase field) for 10 min and oil-quenched to induce the martensitic transformation. After that, the ring subjected to tempering at 1000 °C for 1 h to obtain a stable Q&T microstructure. A number of cylindrical samples with a dimension of Φ8 × 12 mm were machined from the ring for compression tests, with their axes parallel to that of the ring. By using the above processing method (see Figure 1), it ensures that all the compression samples have experienced the same thermo-cycles and hence have the same microstructure. The compression tests were performed on a Gleeble thermo-mechanical simulator (DSI, New York, NY, USA) at temperatures of 950 °C, 1000 °C and 1050 °C, and strain rates of 0.005 s^−1^, 0.05 s^−1^ and 0.5 s^−1^. After a height reduction of 60%, the compressed samples were water-quenched to room temperature.

### 2.2. Microstructure Characterization

After compression, the samples were cut along the compression axis and the cross-sections were mechanical polished. The deformed microstructure was examined by electron back-scattered diffraction (EBSD) (Oxford Instruments, Oxford, UK) conducted on a Zeiss-∑IGMA500 scanning electron microscope (Carl Zeiss AG, Oberkochen, Germany). To gain detailed microstructural information, the EBSD as well as the subsequent analysis was carefully performed according to the following methods: (i) The scanning region (with an area of 20 × 15 mm^2^) was located at the center of the samples and also concentrated in one initial β grain (the initial β grain size is quite large), using a step size of 50 nm; (ii) The γ lattice was treated as normal face-centered cubic structure because its slight tetragonality cannot be easily resolved by EBSD [14]; (iii) It was regarded as high angle grain boundary (HAGB) when the misorientation anlge was higher than 10°. Correspondingly, low angle grain boundaries (LAGB) are those with misorientations less than 10°. The twin boundaries (TB, Σ3) in γ phase have also been determined when the misorientation deviation less than 5° from the ideal (<111>/60°); (iv) According to the standard ISO 13067 [15], the cut-off value for grain identification is 10 pixels, namely, a detected grain with more than 10 pixels was regarded as a true grain; (v) The local texture was analyzed by the JTEX software (Version 2.33, Universite de Lorraine, Metz, France) [16].

## 3. Results

### 3.1. Inital Microstructure

**Figure 2:**
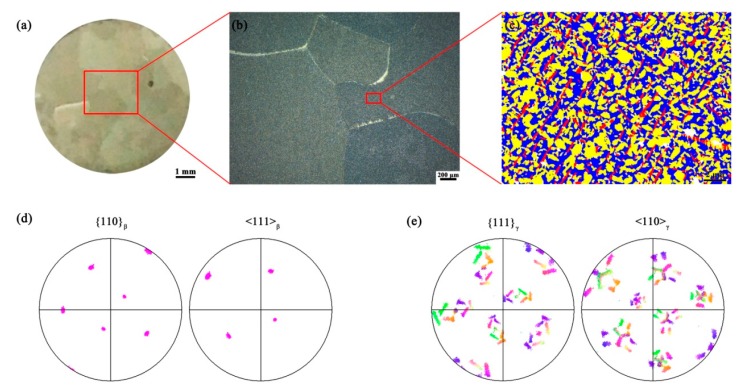
(**a**) Macrostructure of the etched cross-section of the sample prior to compression; (**b**) Optical image of the Q&T microstructure. The ultra-large β grains can be clearly observed; (**c**) A local phase map with a dimension of 20 × 15 μm reveals that the microstructure consists of three phases: β in blue, α_2_ in red and γ in yellow; (**d**,**e**) are the pole figures for β and γ phase, respectively. Note that a Kurdjumov-Sachs (K-S) orientation relationship is evident.

The Q&T microstructure of the Ti-40Al-10V alloy is shown in Figure 2. From the macro-photograph (Figure 2a) and the optical image (Figure 2b) one can note that the alloy is mainly consisted of ultra-large β grains with a mean size of ~2 mm. However, the phase map shown in Figure 2c indicates that there are numerous γ laths and a small number of α_2_ laths existed in the β matrix. The formation mechanism of the Q&T microstructure has been analyzed elsewhere [12] and therefore is briefly delineated here. During annealing at single β phase field, the β grains rapidly grow up due to the ultra-high temperature as well as the lack of a second phase restricting the excessive growth. Therefore, an ultra-coarse β grain structure is produced. During quenching, an incomplete β→α_2_′ martensitic transformation occurs so that ~60 vol % β phase has transformed into α_2_′ lath martensite according to a strict Burgers orientation relationship ({110}_β_//{00.1}_α_ and <111>_β_//<11.0>_α_). After tempering, most of the α_2_′ phase was decomposed into submicrocrystalline γ laths following the Blackburn orientation relationship ({00.1}_α_//{111}_γ_ and <11.0>_α_//<110>_γ_). Consequently, there is a definite Kurdjumov-Sachs (K-S) orientation relationship between β and γ. The K-S orientation relationship as well as the twenty-four γ variants is clearly manifested by the pole figures shown in Figure 2d,e. Note that because the scanning region is within a single β grain, there is only one orientation for the β phase.

### 3.2. Deformation Kinetics

**Figure 3:**
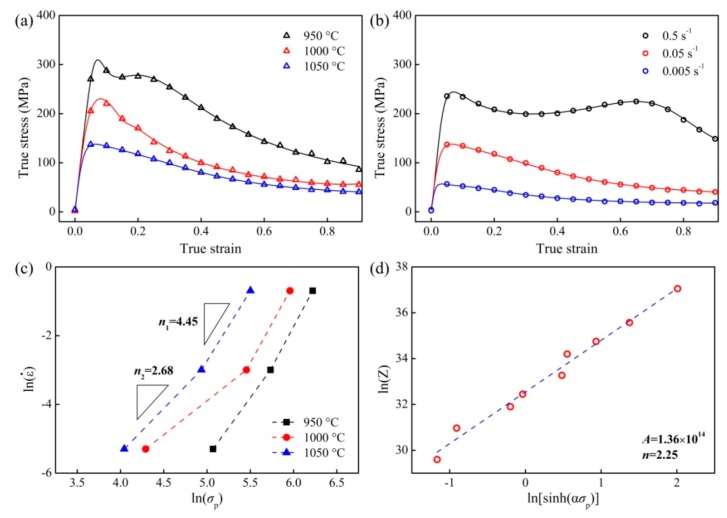
Flow curves at (**a**) 0.05 s^−1^ and (**b**) 1050 °C for the present alloy; (**c**) Variation of the stress exponent with stress; (**d**) Linear relationship between Zener–Hollomon parameter and peak stress.

The true stress-strain curves at various temperatures and strain rates are shown in Figure 3a,b. One can observe that the present flow curves are analogous to those of ordinary TiAl alloys, which are characterized by a single peak at the initial stage and then continuously soften to reach a steady-state flow. However, the flow curve at 0.5 s^−1^ exhibits secondary hardening after a transient softening stage, indicating a variation in deformation mechanisms. Such a transition can be reflected by the evident variation of the stress exponent. As shown in Figure 3c, the *n* value is about 4.5 at high strain rate regime but it is only ~2.7 at low strain rates.

Based on the hyperbolic-sine equation mentioned in Section 1, the relationship between the peak stress and the strain rate can be accurately described, as shown in Figure 3d:

(2) $$\dot{\varepsilon }\exp\left(384\;000/RT\right)=1.36\times 10^{14}{\left[\sinh\left(0.0054\sigma \right)\right]}^{2.25},$$

Interestingly, although there is a large volume fraction of β phase in the present alloy, the apparent activation energy is as large as 384 kJ/mol, which is actually close to the Al self-diffusion energy in the γ-TiAl lattice. Meanwhile, the calculated stress exponent is much lower than those of the ordinary β-lean TiAl alloys. One should note that such a result (high *Q*_app_ but low *n*) is consistent with other studies regarding the V-rich TiAl alloys [10,11].

### 3.3. Microstructure Evolution

#### 3.3.1. Strain Rate Effects

**Figure 4:**
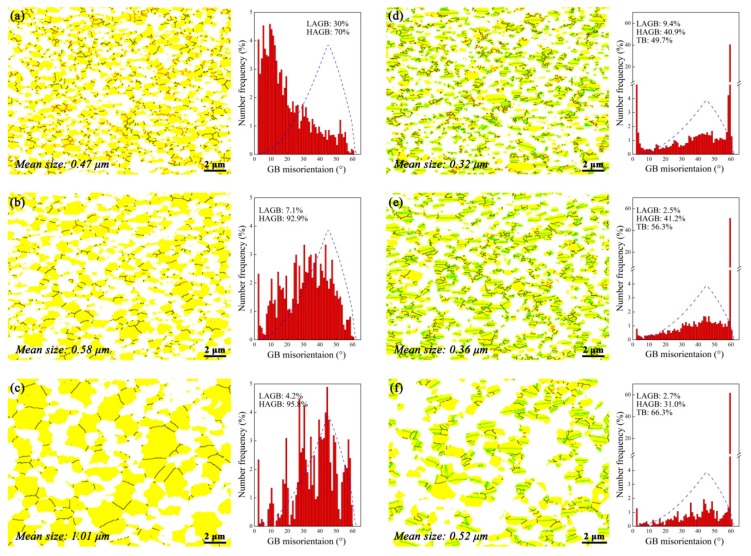
Grain boundary maps and the corresponding grain boundary distribution histogram for (**a**–**c**) β and (**d**–**f**) γ phase at 1050 °C under (**a**,**d**) 0.5 s^−1^; (**b**,**e**) 0.05 s^−1^ and (**c**,**f**) 0.005 s^−1^. The solid back and red lines denote the high angle grain boundary (HAGB) and low angle grain boundary (LAGB), respectively. The twin boundaries (TB) are in blue. The mean grain size is also tabulated on the maps. The dash blue lines represent the random distribution of grain boundary distribution. The compression axis is vertical.

The characteristics of the deformed microstructure can be clearly delineated by the grain boundary (GB) maps. The GB distribution of the present alloy after compression at 1050 °C under different strain rates are shown in Figure 4. It should be stressed again that for each map the scanning region is concentrated in one primary β grain. At first glance one can observe that evident dynamic recrystallization (DRX) occurs in the β matrix at all conditions, which leads to the fragmentation of the initially large β grain, as clearly manifested by the GB maps shown in Figure 4a–c. However, it lacks heterogeneous DRX nuclei as a typical indication of conventional discontinuous DRX [17]. In contrast, the β grain structure evolves in a homogeneous and gradual manner, i.e., a direct transition from LAGB to HAGB by persistent absorption of lattice dislocations. This is a clear evidence of continuous DRX, or in other words, in-situ recrystallization/extended recovery [17]. When deformed at 0.5 s^−1^, the DRX process is not complete due to the fast straining so that ~30% LAGB is retained. As the strain rate decreasing, the number fraction of LAGB is dramatically reduced and almost disappear at the lowest strain rate (Figure 4c), indicating a full-DRXed and stable grain structure at such a condition. Meanwhile, the DRXed grain size is abruptly increased at the lowest strain rate, which cannot be simply clarified by the lower strain rate. Anyway, all the evidence indicates that the β grain structure is significantly affected by the strain rate.

As for the γ phase, three distinct features can be concluded from the GB maps. Firstly, at high strain rates (e.g., Figure 4d), the γ phase remains a lath-like morphology, but tends to aligns perpendicular to the compression direction (CD). At the lowest strain rate (Figure 4f) the γ laths are notably spheroidized. This indicates the dramatic occurrence of strain-induced interface migration at low strain rates. Secondly, the TB number fraction remains a very high level despite different strain rates, and becomes even higher as the strain rate increasing. At the lowest strain rate (Figure 4f), the TB fraction is as high as 66%. Thirdly, under the 0.5 s^−1^ strain rate (Figure 4d) there is only 9% LAGB in the deformed sample, whereas the LAGB density is further decreased with the decreasing strain rates, and is even rarely observed at the lowest strain rate.

**Figure 5:**
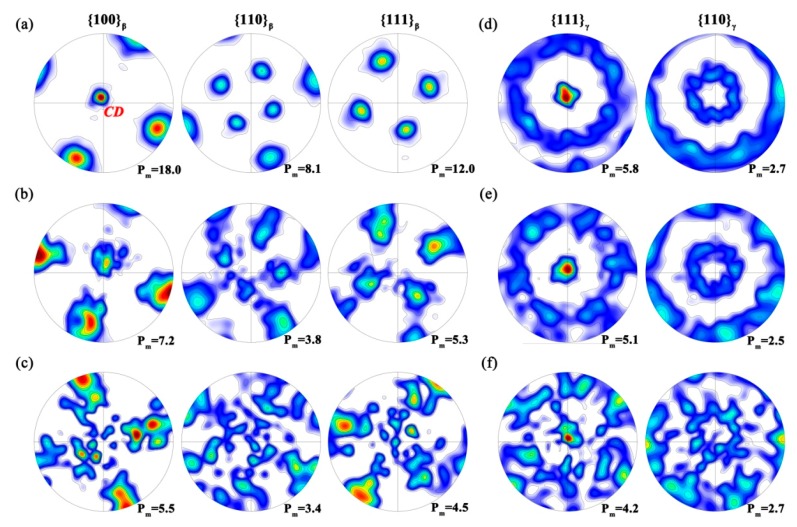
Pole figurs for (**a**–**c**) β and (**d**–**f**) γ phase at 1050 °C under (**a**,**d**) 0.5 s^−1^; (**b**,**e**) 0.05 s^−1^ and (**c**,**f**) 0.005 s^−1^. The maximum intensity is listed in the bottom-right for each pole figure.

Another interesting phenomenon regarding the interaction between β and γ is the enhancing “wetting” of the γ GB with decreasing strain rates. That is, the soft β matrix tends to penetrate along some γ GB and separate the γ clusters. This can be evidently demonstrated by comparison among Figure 5d–f where the γ clusters become more discrete with decreasing strain rates. The GB wetting for various well-annealed alloys has been studied in a number of previous works (e.g., [18] and references therein). Take the present alloy, for example, if the β/γ interface energy is lower than that of the γ GB, then the β tends to increase its surface via wetting the γ GB during annealing. Considering the deformation conditions with excessive lattice dislocations, it does not promote the formation of well-organized low-energy interfaces [19] and hence the conventional GB wetting is not readily observed. In contrast, under superplastic deformation condition where the recovery process extensively occurs, the wetting-like phenomenon is evident in some alloys. For instance, Zherebtsov et al. [20] noted that during superplastic deformation of a Ti-6Al-4V alloy the initially discrete β particles spread along the α/α GB and formed very thin layers. Such a configuration resembling a soft lubricating layer plays a significant role in improving ductility, limiting cavitation and microstructural coarsening during superplastic deformation [20,21]. Therefore, in the present case the enhancing γ GB “wetting” with decreasing strain rate may be an indication of increasing grain boundary sliding (GBS).

The pole figures corresponding to the maps in Figure 4 are shown in Figure 5. One should note that these pole figures do not represent the real texture because each EBSD map is concentrated within a single initial β grain. Therefore, they actually delineate the “orientation fragmentation” of a certain β grain. At the first glance, an impressive phenomenon is that, as shown in Figure 5a–c, at each strain rate the β orientations coincidently rotate and align their <100>_β_ axes parallel to CD. Therefore, a preferential β orientation resembling the <100> fiber tends to be formed. Meanwhile, no other preferential component can be observed. In general, the typical texture produced in compressed body-centered cubic (BCC) metallic materials is <100> + <111> fibers [22,23]. With the decreasing *Z* value, i.e., temperature increase and strain rate decrease, the <100> intensity is increased while the <111> becomes weak. Indeed, the formation of the preferential β orientation in the present case is readily clarified by the traditional theory described above. However, one should note that the poles become diffused as the strain rate decreasing, which results in degraded intensity maxima. This is contradictory to the fact that low *Z* value (increasing temperature and decreasing strain rates) can strengthen the <100> component [23]. Moreover, the newly formed <100> orientation tends to be vanishing at the lowest strain rate. This is a strong evidence that the GBS becomes prominent [24]. Detailed discussion will be proposed later.

As for the γ phase, the pole figures shown in Figure 5d–f manifest that, firstly, the definite K-S orientation relationship between β and γ is completely destroyed. This is a natural consequence because an orientation relationship can be readily destroyed by many factors such as intragranular deformation, DRX, GBS, etc. Secondly, <111>_γ_ aligned to CD is observed, and thereby a single <111>_γ_ fiber is produced. However, for ordinary (α_2_ + γ) TiAl alloys, in general, the texture components produced during uniaxial compression include <110]_γ_ and <302]_γ_, as well as the <100]_γ_ fiber caused by DRX [25,26,27]. If analyzed as cubic system, these texture components would be <110>_γ_ and <100>_γ_ instead [24], which are identical with those in normal FCC materials [25]. Therefore, the <111>_γ_ fiber formed in the present case is impossibly caused by intragranular deformation. Thirdly, as the decreasing strain rate, the pole intensity is continuously weakened. At the lowest strain rate, the <111>_γ_ fiber tends to be decomposed as that in the β phase.

#### 3.3.2. Temperature Effects

**Figure 6:**
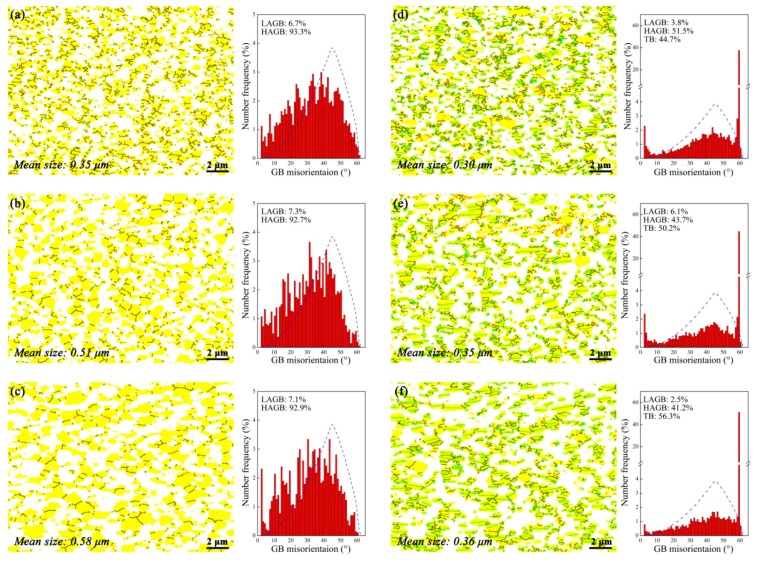
Grain boundary maps and the corresponding grain boundary distribution histogram for (**a**–**c**) β and (**d**–**f**) γ phase at (**a**,**d**) 950 °C; (**b**,**e**) 1000 °C and (**c**,**f**) 1050 °C, under a constant strain rate of 0.05 s^−1^. The solid back and red lines denote the high angle grain boundary (HAGB) and low angle grain boundary (LAGB), respectively. The twin boundaries (TB) are in blue. The dash blue lines represent the random distribution of grain boundary distribution. The mean grain size is also tabulated on the maps. The compression axis is vertical.

Figure 6 is the GB characteristics of the alloy after deformation at 0.05 s^−1^ under different temperatures. One can note that for the β matrix (Figure 6a–c), the LAGB is in a fairly low density at all temperatures, indicating an almost complete DRX. Meanwhile, the DRXed grain size is slightly increased with the increasing temperature. For the γ phase (Figure 6d–f), with the elevating temperature, the TB fraction is slightly increased accompanied by the slight decrease of the HAGB, whereas the LAGB remains a low number fraction. In conclusion, it is interesting to note that the deformation temperature plays an insignificant role in the microstructure evolution, in comparison to the strain rate.

**Figure 7:**
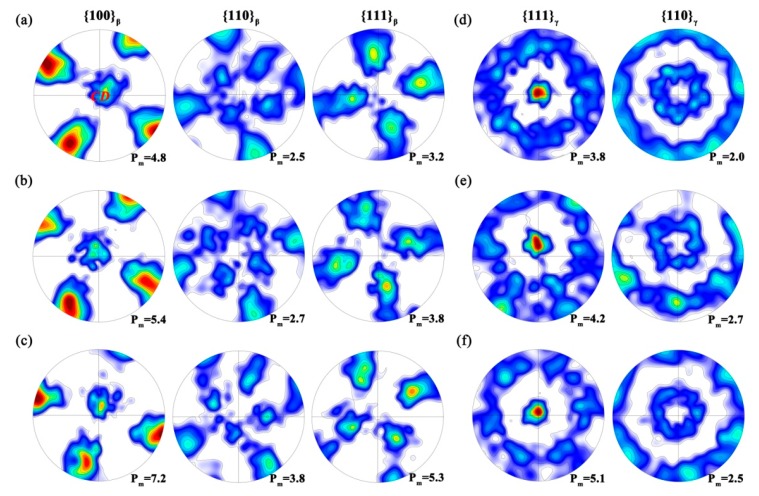
Pole figures for (**a**–**c**) β and (**d**–**f**) γ phase at (**a**,**d**) 950 °C; (**b**,**e**) 1000 °C and (**c**,**f**) 1050 °C, under a constant strain rate of 0.05 s^−1^.. The maximum intensity is listed in the bottom-right for each pole figure.

Similar observations can also been noticed in the pole figures. As shown in Figure 7, for all the strain rates a <100>_β_ and <111>_γ_ “fiber texture” are respectively formed in the β and γ phase, as those in Figure 5. The temperature increase makes no apparent difference in the pole figure morphology. Only the pole intensity is slightly strengthened by the increasing temperature. This observation, however, seems to be contradictory to the common sense that the GBS contribution is increased with increasing temperature. A more detailed analysis will be carried out later.

## 4. Discussion

### 4.1. Deformation Mechanisms

In general, the intragranular deformation is predominant during the dislocation creep of metallic materials including ordinary (α_2_ + γ) TiAl alloys, for either the power-law creep or the power-law breakdown regime. However, the deformation behavior of the present alloy reflects some unexpected features from both kinetics and microstructure aspects, indicating unique deformation mechanisms.

Strain rate plays a significant role during hot deformation. Under the highest strain rate, it is found that extensive intragranular deformation occurs within the β matrix, which results in the evident occurrence of continuous DRX and a high density of LAGB (Figure 4a). Because the deformation temperature is sufficiently high, a single <100>_β_ fiber is produced via the synergic effect of strain-induced GB migration and continuous DRX [22]. In contrast, the γ phase is expected to participate much less deformation in comparison with the soft β matrix because it is much harder than the soft β phase at elevated temperature. Hence the LAGB remains a fairly low density in the γ laths. Meanwhile, as manifested by Figure 4d, the γ laths concurrently rotate toward a “hard orientation” during straining, namely, the γ laths align perpendicularly to the CD. The formation of such a “hard orientation” seems analogous to that in the (α_2_ + γ) TiAl alloys where the remnant α_2_/γ lamellar colonies aligned perpendicular to the deformation direction [28]. According to our previous study [12], the formation mechanism of the γ laths in the Q&T microstructure is crystallographically the same with that in ordinary α_2_/γ lamellae. Therefore, it may be speculated that the habit plane for the present γ laths is still {111}_γ_ [29]. As a consequence of the lath rotation, the <111>_γ_ orientations align parallel to the CD, and hence a single {111}_γ_ fiber is produced rather than the typical deformation texture. In addition, by using the proposed mechanism, i.e., deformation is mainly concentrated within β matrix accompanied by γ lath rotation, can also clarify the secondary hardening of the flow curve at highest strain rate (Figure 3b) to some extent. That is, the γ laths with hard orientation unquestionably prevent the further rotation and leads to the subsequent deformation more difficult.

As the strain rate decreasing, the contribution of GBS and β/γ interface sliding on the deformation becomes prominent, which results in the vanishing of the preferential orientations (Figure 5c,f). Meanwhile, stimulated by the enhanced GBS as well as the interface sliding, apparent strain-induced grain growth (Figure 4c) and dramatic interface migration (results in the notable globalization of the submicrocrystalline γ laths, as shown in Figure 4f) are observed. Note the strain-induced globalization and strain-induced grain growth are consistent with those during superplastic tension of fine-grained metallic materials [30,31]. All these microstructural evidence, as well as the low stress exponent (Figure 3c), indicates that the major deformation mechanism is GBS plus interface sliding at the lowest strain rate. In addition, numerous annealing twins are formed during the globalization process of γ laths (as manifested in Figure 4f), leading to an increasing TB fraction. However, it should be pointed out that accommodation processes are required to release the stress concentrations at triple-junctions caused by GBS or interface sliding, and the major one would be rate-controlling. In the present case, it is readily deduced that the rate-controlling step at lowest strain rate regime is still concentrated within the β phase, namely, the local stress concentration can be relieved by dislocation climb/glide in the soft β matrix [32].

In comparison with strain rate, temperature has an insignificant effect on the deformation behavior. As pointed out in Section 3.3.2, the microstructure, as well as the local texture, are slightly affected by the temperature, but a curious observation is that the local texture is somewhat strengthened with the temperature increase. This can be rationalized as follows. Under the current strain rate level (0.05 s^−1^), the predominant deformation mechanism is the intragranular deformation of β phase at all temperatures. The GBS, as well as the interface sliding, plays an insignificant role. However, the increasing temperature causes the strain-induced GB migration more readily and therefore the <100>_β_ intensity is increased. Simultaneously, the rotation process of the γ laths is also accelerated by the increasing temperature, which leads to an increasing <111>_γ_ intensity.

In conclusion, the deformation mechanism of the present alloy is significantly affected by strain rate rather than temperature in the testing range. At high strain rate, the major deformation mechanism is the intragranular deformation (i.e., dislocation creep) in the β matrix, whereas the GBS and interface sliding are predominant in the low *Z* regime. For all the deformation conditions, the rate-controlling mechanism is believed to be concentrated in the soft β phase. All the microstructure observations, as well as the change of the stress exponent (Figure 3c), can be clarified by the proposed mechanism. However, the unexpectedly high apparent activation energy (384 kJ/mol) requires further clarification.

### 4.2. On the Hyperbolic-Sine Law

As pointed out in the Introduction, the hyperbolic-sine law (Equation (1)) is widely used to describe the deformation kinetics of metallic materials, because it describes the dislocation creep behavior in the power-law and power-law breakdown regimes quite accurate [33]. However, it should be stressed that it is a phenomenological model and has little theoretical basis. In comparison with the physically based power-law equation [34], i.e.,

(3) $$\dot{\varepsilon }\exp\left(Q_{D}/RT\right)=A\sigma^{n},$$

There are two mathematical differences. Firstly, in the hyperbolic-sine law the *σ* item is replaced by the sinh(*ασ*) item in order to straighten the stress-strain rate curves in a logarithmic scale (in the power-law breakdown regime, the stress exponent is progressively increased with the *Z* value). Secondly, the *Q*_app_ in the hyperbolic-sine law is an empirical variable which is just an approximation of the effective diffusion energy (*Q*_D_ in the power-law equation). Here we use a simple example to reveal the application conditions for the hyperbolic-sine law. 

The applied dislocation creep data of pure Al is quoted from Ref. [35]. This creep dataset shows a clear transition from power-law creep to power-law breakdown. Comparisons are performed to examine the variation of the activation energy and stress exponent with creep conditions. If uses the entire dataset, it is obtained that *Q*_app_ is 153 kJ/mol, and the corresponding *n* value is 4.0. If uses the data at low stresses (power-law creep regime), the calculated *Q*_app_ and *n* value are 152 kJ/mol and 5.0, respectively. Concurrently, in the high-stress regime (power-law breakdown) the *n* is about 7.1 whereas the *Q*_app_ value is nearly unchanged (151 kJ/mol). Therefore, one can conclude that the *Q*_app_ value is almost independent on the applied stress from the power-law creep to power-law breakdown. This is believed to be a key factor for the application of the hyperbolic-sine law. Such a conclusion is also valid for (α_2_ + γ) TiAl alloys, where the apparent activation energy for the power-law breakdown is 370 kJ/mol and is nearly equal to that of power-law creep [6].

In contrast, for the present alloy, it is not the case of dislocation creep but shows a transition from GBS to dislocation creep as mentioned above. In the high strain rate regime the re-estimated *Q*_app_ value is about 325 kJ/mol, while in the low strain rate regime it is only 245 kJ/mol. Note that both values are much lower than that calculated by using the entire dataset (384 kJ/mol as obtained in Section 3.2). Therefore, it can be concluded that the hyperbolic-sine law is invalid on rationalizing the deformation kinetics when it is not the case of dislocation creep, especially there is a transition in rate-controlling process. Then the obtained *Q*_app_ based on the entire dataset seems meaningless. Furthermore, for either the 325 kJ/mol or the 245 kJ/mol, it is much lower than the activation energy of dislocation/superplastic creep for the (α_2_ + γ) TiAl alloys (360 kJ/mol [6]), i.e., Al self-diffusion energy in TiAl lattice. Therefore, it can be deduced that the rate-controlling mechanism of the present case, for both the dislocation creep at high strain rates and the GBS at low strain rates, is concentrated in the soft β matrix, rather than in the γ phase. Such a speculation is consistent with that discussed in Section 4.1.

## 5. Conclusions

In this study, the deformation behavior of a Ti-40Al-10V alloy with Q&T microstructure has been examined by isothermal compression at various temperature and strain rates. It was found that, despite the ultra-coarse grain structure, the present alloy with a Q&T microstructure exhibited superplastic flow even at industrial wrought conditions, whereby the hot workability was expected to be significantly improved considering the natural brittleness of TiAl alloys. Such an alloy & microstructure design may provide a new strategy for fabricating complex-shaped but cost-effective TiAl products. The main conclusions for the present study are drawn below:

1. The Q&T microstructure of the Ti-40Al-10V alloy mainly consisted of ultra-large β grains with a mean size of ~2 mm and numerous γ laths in the β matrix. Only a small number of α_2_ laths existed. A definite Kurdjumov-Sachs orientation relationship was identified between β and γ phase.
2. The flow curves were characterized by a single peak at the initial stage and then continuously soften to reach a steady-state flow. However, the flow curve at 0.5 s^−1^ exhibited a secondary hardening stage after a transient softening stage. Based on the hyperbolic-sine law, the estimated apparent activation energy (*Q*_app_) was 384 kJ/mol, and the stress exponent value was determined to be ~2.25. Detailed analysis revealed that the *Q*_app_ values were 325 kJ/mol and 245 kJ/mol at high and low strain rate range, respectively, with corresponding stress exponent values of 2.7 and 4.5. The hyperbolic-sine law was believed to be invalid when it was not the case of dislocation creep, especially there was a transition in rate-controlling process.
3. For all the deformed samples, continuous dynamic recrystallization intensively occurred in the β matrix, accompanied by the simultaneous rotation of the γ laths. Meanwhile, a preferential orientation of <100>_β_ and <111>_γ_ parallel to the compression axis was observed for β and γ phase, respectively. With the decreasing strain rates, the grain boundary/interface sliding gradually became prominent which resulted in some superplastic deformation features such as intensive strain-induced grain growth and interface migration, as well as continuous weakening/vanishing of the local texture, etc. Meanwhile, the temperature had slight effects on deformation behavior. After a detailed discussion, it was believed that the deformation mechanism was the intragranular deformation (or dislocation creep) in the β matrix under high strain rates, but gradually evolved to the grain boundary/interface sliding mechanism at low strain rate regime.
